# 4-Methyl-5-phenyl-1*H*-pyrazol-3(2*H*)-one

**DOI:** 10.1107/S160053681005213X

**Published:** 2010-12-18

**Authors:** Wan-Sin Loh, Hoong-Kun Fun, R. Venkat Ragavan, V. Vijayakumar, S. Sarveswari

**Affiliations:** a X-ray Crystallography Unit, School of Physics, Universiti Sains Malaysia, 11800 USM, Penang, Malaysia; b Organic Chemistry Division, School of Advanced Sciences, VIT University, Vellore 632 014, India

## Abstract

The asymmetric unit of the title compound, C_10_H_10_N_2_O, contains two crystallographically independent mol­ecules with similar geometries, which exist in the keto form. The C=O bond lengths are 1.2878 (12) Å in mol­ecule *A* and 1.2890 (12) Å in mol­ecule *B*, indicating that the compound undergoes enol-to-keto tautomerism during the crystallization process. In mol­ecule *A*, the pyrazole ring is approximately planar [maximum deviation = 0.007 (1) Å] and forms a dihedral angle of 36.67 (6)° with the attached phenyl ring. In mol­ecule *B*, the dihedral angle formed between the pyrazole ring [maximum deviation = 0.017 (1) Å] and the phenyl ring is 41.19 (6)°. In the crystal, inter­molecular N—H⋯O hydrogen bonds link neighbouring mol­ecules into dimers generating *R*_2_^2^(8) ring motifs. These dimers are linked into ribbons along [101] *via* inter­molecular N—H⋯O hydrogen bonds, forming *R*_4_^2^(10) ring motifs.

## Related literature

For background to pyrazole derivatives and their anti­microbial activity, see: Ragavan *et al.* (2009 ▶, 2010 ▶). For bond-length data, see: Allen *et al.* (1987 ▶). For the structure of the enol form of this mol­ecule, see: Shahani *et al.* (2010 ▶). For other related structures, see: Loh *et al.* (2010**a* ▶,*b* ▶,c* ▶). For hydrogen-bond motifs, see: Bernstein *et al.* (1995 ▶). For the stability of the temperature controller used in the data collection, see: Cosier & Glazer (1986 ▶).
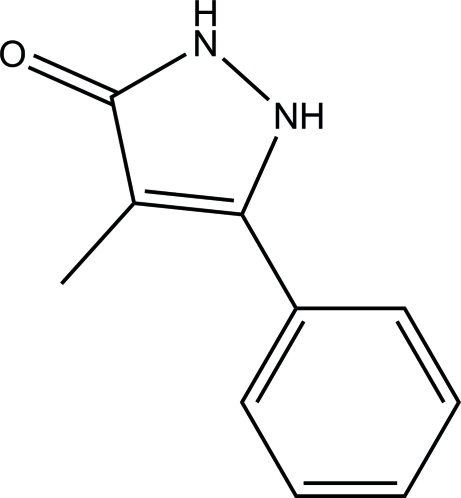


## Experimental

### 

#### Crystal data

C_10_H_10_N_2_O
*M* *_r_* = 174.20
Monoclinic, 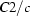

*a* = 25.9337 (4) Å
*b* = 10.8100 (1) Å
*c* = 14.1426 (2) Å
β = 118.961 (1)°
*V* = 3468.98 (8) Å^3^
*Z* = 16
Mo *K*α radiation
μ = 0.09 mm^−1^
*T* = 100 K
0.45 × 0.39 × 0.25 mm

#### Data collection

Bruker SMART APEXII CCD area-detector diffractometer
Absorption correction: multi-scan (*SADABS*; Bruker, 2009 ▶) *T*_min_ = 0.961, *T*_max_ = 0.978
36992 measured reflections
5087 independent reflections
4389 reflections with *I* > 2σ(*I*)
*R*_int_ = 0.036

#### Refinement

*R*[*F* ^2^ > 2σ(*F* ^2^)] = 0.044
*wR*(*F* ^2^) = 0.119
*S* = 1.03
5087 reflections
253 parameters
H atoms treated by a mixture of independent and constrained refinement
Δρ_max_ = 0.45 e Å^−3^
Δρ_min_ = −0.22 e Å^−3^

### 

Data collection: *APEX2* (Bruker, 2009 ▶); cell refinement: *SAINT* (Bruker, 2009 ▶); data reduction: *SAINT*; program(s) used to solve structure: *SHELXTL* (Sheldrick, 2008 ▶); program(s) used to refine structure: *SHELXTL*; molecular graphics: *SHELXTL*; software used to prepare material for publication: *SHELXTL* and *PLATON* (Spek, 2009 ▶).

## Supplementary Material

Crystal structure: contains datablocks global, I. DOI: 10.1107/S160053681005213X/sj5074sup1.cif

Structure factors: contains datablocks I. DOI: 10.1107/S160053681005213X/sj5074Isup2.hkl

Additional supplementary materials:  crystallographic information; 3D view; checkCIF report

## Figures and Tables

**Table 1:** Hydrogen-bond geometry (Å, °)

*D*—H⋯*A*	*D*—H	H⋯*A*	*D*⋯*A*	*D*—H⋯*A*
N1*B*—H1*NB*⋯O1*A*^i^	0.913 (17)	1.796 (17)	2.7001 (11)	170.0 (16)
N1*A*—H1*NA*⋯O1*B*	0.935 (19)	1.78 (2)	2.6987 (14)	165.9 (16)
N2*A*—H2*NA*⋯O1*A*^ii^	0.93 (2)	1.768 (19)	2.6917 (12)	173.9 (17)
N2*B*—H2*NB*⋯O1*B*^iii^	0.934 (18)	1.752 (18)	2.6850 (13)	177.0 (16)

Symmetry codes: (i) 
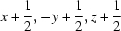
; (ii) 
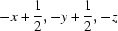
; (iii) 
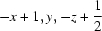
.

**Fig. 1.**
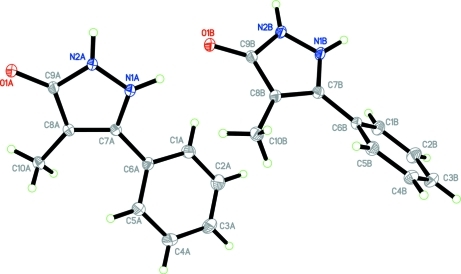
The molecular structure of the title compound, showing 50% probability displacement ellipsoids and the atom-numbering scheme.

**Fig. 2.**
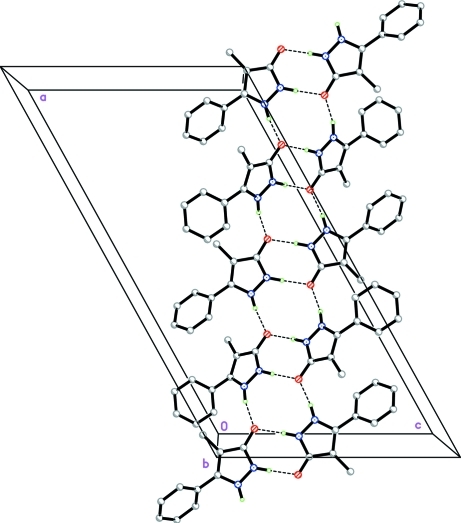
The crystal packing of the title compound, viewed along the b axis. H atoms not involved in the intermolecular interactions (dashed lines) have been omitted for clarity.

## supplementary crystallographic information

### Comment

Antibacterial and antifungal activities of the azoles are most widely studied and some of them are in clinical practice as anti-microbial agents.  However, the azole-resistant strains have led to the development of new anti-microbial compounds. In particular, pyrazole derivatives are extensively studied and used as anti-microbial agents. Pyrazoles represent an important class of heterocyclic compounds and many pyrazole derivatives are reported to have a broad spectrum of biological properties such as anti-inflammatory, antifungal, herbicidal, anti-tumour, cytotoxic, molecular modelling and antiviral activities. Pyrazole derivatives also act as anti-angiogenic agents, A3 adenosine receptor antagonists, neuropeptide YY5 receptor antagonists as well as kinase inhibitors for the treatment of type 2 diabetes, hyperlipidemia, obesity and thrombopiotinmimetics. Recently urea derivatives of pyrazoles have been reported as potent inhibitors of p38 kinase. Since the high electronegativity of halogens (particularly chlorine and fluorine) in the aromatic part of the drug molecules plays an important role in enhancing their biological activity, we are interested to have 4-fluoro or 4-chloro substitution in the aryls of 1,5-diaryl pyrazoles. These properties and applications are discussed in our previous reports on the synthesis of novel pyrazole derivatives and their microbial activities (Ragavan *et al.*, 2009, 2010). The enol-form of this compound has been already reported in the literature (Shahani *et al.*, 2010).

The title compound (Fig. 1), consists of two crystallographically independent molecules, with similar geometries and exists in the keto-form. This indicates that the compound undergoes an enol-to-keto tautomerism during the crystallization process with the bond length of C═O being 1.2878 (12) Å in molecule *A* and 1.2890 (12) Å in molecule *B*. In molecule *A*, the pyrazole ring (N1A/N2A/C7A–C9A) is approximately planar (maximum deviation of 0.007 (1) Å at N1A) and forms a dihedral angle of 36.67 (6)° with the attached phenyl ring (C1A–C6A). In molecule *B*, the dihedral angle formed between the pyrazole ring (N1B/N2B/C7B–C9B) [maximum deviation of 0.017 (1) Å at C9B] and the phenyl ring (C1B–C6B) is 41.19 (6)°. Bond lengths (Allen *et al.*, 1987) and angles are within the normal ranges and are comparable to the related structures (Loh *et al.*, 2010**a*,*b*,c*).

In the crystal packing (Fig. 2), intermolecular N2A—H2NA···O1A and N2B—H2NB···O1B hydrogen bonds (Table 1) link the neighbouring molecules to form dimers, generating *R*_2_^2^(8) ring motifs (Bernstein *et al.*, 1995). These set of dimers are linked into ribbons along the [101], *via* intermolecular N1A—H1NA···O1B and N1B—H1NB···O1A hydrogen bonds (Table 1), forming *R*_4_^2^(10) ring motifs (Bernstein *et al.*, 1995).

### Experimental

The compound was synthesized using a literature method (Ragavan *et al.*, 2009, 2010) and recrystallized from ethanol-chloroform; 1:1. *M. p.*: 493–494 K, yield: 72%.

### Refinement

N– bound H atoms were located from a difference Fourier map and refined freely [N–H = 0.913 (17) to 0.935 (16) Å]. The remaining H atoms were positioned geometrically with bond lengths C–H = 0.93 to 0.96 Å and were refined using a riding model, with *U*_iso_(H) = 1.2 or 1.5 *U*_eq_(C). A rotating group model was applied to the methyl groups.

### Crystal data

**Table:**

C_10_H_10_N_2_O	*F*(000) = 1472
*M**_r_* = 174.20	*D*_x_ = 1.334 Mg m^−^^3^
Monoclinic, *C*2/*c*	Mo *K*α radiation, λ = 0.71073 Å
Hall symbol: -C 2yc	Cell parameters from 9946 reflections
*a* = 25.9337 (4) Å	θ = 2.4–30.1°
*b* = 10.8100 (1) Å	µ = 0.09 mm^−^^1^
*c* = 14.1426 (2) Å	*T* = 100 K
β = 118.961 (1)°	Block, colourless
*V* = 3468.98 (8) Å^3^	0.45 × 0.39 × 0.25 mm
*Z* = 16	

### Data collection

**Table:**

Bruker SMART APEXII CCD area-detector diffractometer	5087 independent reflections
Radiation source: fine-focus sealed tube	4389 reflections with *I* > 2σ(*I*)
graphite	*R*_int_ = 0.036
φ and ω scans	θ_max_ = 30.1°, θ_min_ = 1.8°
Absorption correction: multi-scan (*SADABS*; Bruker, 2009)	*h* = −36→36
*T*_min_ = 0.961, *T*_max_ = 0.978	*k* = −15→15
36992 measured reflections	*l* = −19→18

### Refinement

**Table:**

Refinement on *F*^2^	Primary atom site location: structure-invariant direct methods
Least-squares matrix: full	Secondary atom site location: difference Fourier map
*R*[*F*^2^ > 2σ(*F*^2^)] = 0.044	Hydrogen site location: inferred from neighbouring sites
*wR*(*F*^2^) = 0.119	H atoms treated by a mixture of independent and constrained refinement
*S* = 1.03	*w* = 1/[σ^2^(*F*_o_^2^) + (0.0684*P*)^2^ + 2.050*P*] where *P* = (*F*_o_^2^ + 2*F*_c_^2^)/3
5087 reflections	(Δ/σ)_max_ = 0.001
253 parameters	Δρ_max_ = 0.45 e Å^−^^3^
0 restraints	Δρ_min_ = −0.22 e Å^−^^3^

### Special details

**Table:**

Experimental. The crystal was placed in the cold stream of an Oxford Cryosystems Cobra open-flow nitrogen cryostat (Cosier & Glazer, 1986) operating at 100.0 (1) K.
Geometry. All e.s.d.'s (except the e.s.d. in the dihedral angle between two l.s. planes) are estimated using the full covariance matrix. The cell e.s.d.'s are taken into account individually in the estimation of e.s.d.'s in distances, angles and torsion angles; correlations between e.s.d.'s in cell parameters are only used when they are defined by crystal symmetry. An approximate (isotropic) treatment of cell e.s.d.'s is used for estimating e.s.d.'s involving l.s. planes.
Refinement. Refinement of *F*^2^ against ALL reflections. The weighted *R*-factor *wR* and goodness of fit *S* are based on *F*^2^, conventional *R*-factors *R* are based on *F*, with *F* set to zero for negative *F*^2^. The threshold expression of *F*^2^ > σ(*F*^2^) is used only for calculating *R*-factors(gt) *etc*. and is not relevant to the choice of reflections for refinement. *R*-factors based on *F*^2^ are statistically about twice as large as those based on *F*, and *R*- factors based on ALL data will be even larger.

### Fractional atomic coordinates and isotropic or equivalent isotropic displacement parameters (Å^2^)

**Table:**

	*x*	*y*	*z*	*U*_iso_*/*U*_eq_	
O1A	0.19371 (3)	0.15583 (7)	0.01430 (6)	0.01628 (16)	
N1A	0.33170 (4)	0.19356 (8)	0.23566 (7)	0.01429 (17)	
N2A	0.28884 (4)	0.22212 (8)	0.13372 (7)	0.01378 (17)	
C1A	0.40804 (5)	0.05241 (11)	0.43463 (9)	0.0198 (2)	
H1AA	0.4262	0.0681	0.3931	0.024*	
C2A	0.44188 (5)	0.02010 (12)	0.54257 (10)	0.0249 (2)	
H2AA	0.4826	0.0147	0.5730	0.030*	
C3A	0.41532 (5)	−0.00418 (11)	0.60540 (10)	0.0222 (2)	
H3AA	0.4382	−0.0251	0.6779	0.027*	
C4A	0.35439 (5)	0.00296 (10)	0.55947 (9)	0.0209 (2)	
H4AA	0.3364	−0.0139	0.6012	0.025*	
C5A	0.32019 (5)	0.03512 (10)	0.45141 (9)	0.0188 (2)	
H5AA	0.2794	0.0391	0.4211	0.023*	
C6A	0.34665 (4)	0.06155 (9)	0.38784 (8)	0.01417 (19)	
C7A	0.31075 (4)	0.10488 (9)	0.27595 (8)	0.01305 (19)	
C8A	0.25384 (4)	0.07360 (9)	0.19815 (8)	0.01399 (19)	
C9A	0.24055 (4)	0.14984 (9)	0.10712 (8)	0.01324 (19)	
C10A	0.21385 (4)	−0.02449 (10)	0.20107 (9)	0.0171 (2)	
H10A	0.2367	−0.0958	0.2385	0.026*	
H10B	0.1855	−0.0472	0.1286	0.026*	
H10C	0.1937	0.0066	0.2380	0.026*	
O1B	0.43937 (3)	0.28341 (7)	0.28820 (6)	0.01641 (16)	
N1B	0.58492 (4)	0.29961 (8)	0.48713 (7)	0.01537 (18)	
N2B	0.54157 (4)	0.29083 (8)	0.38216 (7)	0.01525 (18)	
C1B	0.64581 (5)	0.25037 (10)	0.72121 (9)	0.0177 (2)	
H1BA	0.6549	0.1929	0.6825	0.021*	
C2B	0.68154 (5)	0.26106 (11)	0.83201 (9)	0.0225 (2)	
H2BA	0.7144	0.2102	0.8676	0.027*	
C3B	0.66848 (5)	0.34762 (12)	0.89026 (10)	0.0233 (2)	
H3BA	0.6926	0.3549	0.9645	0.028*	
C4B	0.61925 (5)	0.42316 (11)	0.83712 (9)	0.0208 (2)	
H4BA	0.6107	0.4816	0.8758	0.025*	
C5B	0.58278 (5)	0.41160 (10)	0.72656 (9)	0.0168 (2)	
H5BA	0.5494	0.4610	0.6916	0.020*	
C6B	0.59605 (4)	0.32598 (9)	0.66741 (8)	0.01363 (19)	
C7B	0.55927 (4)	0.31664 (9)	0.54975 (8)	0.01354 (19)	
C8B	0.49830 (4)	0.31808 (9)	0.48467 (8)	0.01383 (19)	
C9B	0.48786 (4)	0.29746 (9)	0.37751 (8)	0.01359 (19)	
C10B	0.45201 (4)	0.34052 (10)	0.51676 (9)	0.0184 (2)	
H10D	0.4627	0.2991	0.5838	0.028*	
H10E	0.4149	0.3091	0.4617	0.028*	
H10F	0.4487	0.4277	0.5254	0.028*	
H1NB	0.6234 (7)	0.3100 (16)	0.5038 (13)	0.034 (4)*	
H1NA	0.3696 (7)	0.2271 (15)	0.2649 (13)	0.030 (4)*	
H2NA	0.2954 (8)	0.2691 (17)	0.0857 (14)	0.042 (5)*	
H2NB	0.5490 (7)	0.2858 (15)	0.3239 (13)	0.032 (4)*	

### Atomic displacement parameters (Å^2^)

**Table:**

	*U*^11^	*U*^22^	*U*^33^	*U*^12^	*U*^13^	*U*^23^
O1A	0.0111 (3)	0.0236 (4)	0.0122 (4)	−0.0017 (3)	0.0041 (3)	0.0009 (3)
N1A	0.0108 (4)	0.0179 (4)	0.0116 (4)	−0.0012 (3)	0.0034 (3)	0.0017 (3)
N2A	0.0099 (4)	0.0182 (4)	0.0109 (4)	−0.0012 (3)	0.0031 (3)	0.0018 (3)
C1A	0.0151 (5)	0.0252 (5)	0.0193 (6)	0.0041 (4)	0.0084 (4)	0.0052 (4)
C2A	0.0156 (5)	0.0334 (6)	0.0210 (6)	0.0065 (4)	0.0051 (4)	0.0075 (5)
C3A	0.0239 (5)	0.0223 (5)	0.0155 (5)	0.0037 (4)	0.0057 (4)	0.0052 (4)
C4A	0.0249 (5)	0.0220 (5)	0.0180 (5)	0.0000 (4)	0.0122 (5)	0.0030 (4)
C5A	0.0163 (5)	0.0224 (5)	0.0179 (5)	−0.0006 (4)	0.0083 (4)	0.0020 (4)
C6A	0.0138 (4)	0.0135 (4)	0.0135 (5)	0.0002 (3)	0.0053 (4)	0.0003 (3)
C7A	0.0117 (4)	0.0148 (4)	0.0128 (5)	0.0005 (3)	0.0061 (4)	0.0005 (3)
C8A	0.0121 (4)	0.0157 (4)	0.0143 (5)	−0.0006 (3)	0.0065 (4)	−0.0001 (3)
C9A	0.0102 (4)	0.0165 (4)	0.0128 (5)	−0.0005 (3)	0.0054 (4)	−0.0010 (3)
C10A	0.0145 (4)	0.0183 (5)	0.0176 (5)	−0.0039 (3)	0.0071 (4)	0.0001 (4)
O1B	0.0108 (3)	0.0237 (4)	0.0127 (4)	−0.0020 (3)	0.0041 (3)	−0.0001 (3)
N1B	0.0097 (4)	0.0232 (4)	0.0108 (4)	−0.0011 (3)	0.0030 (3)	−0.0021 (3)
N2B	0.0110 (4)	0.0226 (4)	0.0110 (4)	−0.0009 (3)	0.0044 (3)	−0.0014 (3)
C1B	0.0163 (4)	0.0181 (5)	0.0158 (5)	0.0025 (4)	0.0056 (4)	0.0000 (4)
C2B	0.0198 (5)	0.0258 (5)	0.0163 (6)	0.0041 (4)	0.0042 (4)	0.0042 (4)
C3B	0.0227 (5)	0.0324 (6)	0.0121 (5)	−0.0028 (4)	0.0062 (4)	−0.0006 (4)
C4B	0.0222 (5)	0.0254 (5)	0.0177 (5)	−0.0039 (4)	0.0119 (4)	−0.0056 (4)
C5B	0.0151 (4)	0.0192 (5)	0.0160 (5)	0.0003 (3)	0.0073 (4)	−0.0017 (4)
C6B	0.0121 (4)	0.0155 (4)	0.0118 (5)	−0.0014 (3)	0.0046 (4)	−0.0004 (3)
C7B	0.0127 (4)	0.0142 (4)	0.0130 (5)	0.0005 (3)	0.0056 (4)	−0.0004 (3)
C8B	0.0123 (4)	0.0157 (4)	0.0131 (5)	0.0001 (3)	0.0058 (4)	0.0002 (3)
C9B	0.0116 (4)	0.0141 (4)	0.0146 (5)	−0.0004 (3)	0.0060 (4)	0.0006 (3)
C10B	0.0141 (4)	0.0243 (5)	0.0182 (5)	−0.0004 (4)	0.0089 (4)	−0.0020 (4)

### Geometric parameters (Å, °)

**Table:**

O1A—C9A	1.2878 (12)	O1B—C9B	1.2890 (12)
N1A—C7A	1.3560 (13)	N1B—C7B	1.3533 (13)
N1A—N2A	1.3628 (12)	N1B—N2B	1.3640 (12)
N1A—H1NA	0.935 (16)	N1B—H1NB	0.913 (17)
N2A—C9A	1.3655 (12)	N2B—C9B	1.3641 (12)
N2A—H2NA	0.928 (19)	N2B—H2NB	0.933 (17)
C1A—C2A	1.3875 (16)	C1B—C2B	1.3861 (16)
C1A—C6A	1.4006 (14)	C1B—C6B	1.4004 (14)
C1A—H1AA	0.9300	C1B—H1BA	0.9300
C2A—C3A	1.3878 (17)	C2B—C3B	1.3926 (17)
C2A—H2AA	0.9300	C2B—H2BA	0.9300
C3A—C4A	1.3896 (16)	C3B—C4B	1.3901 (17)
C3A—H3AA	0.9300	C3B—H3BA	0.9300
C4A—C5A	1.3893 (16)	C4B—C5B	1.3866 (16)
C4A—H4AA	0.9300	C4B—H4BA	0.9300
C5A—C6A	1.3994 (14)	C5B—C6B	1.3979 (14)
C5A—H5AA	0.9300	C5B—H5BA	0.9300
C6A—C7A	1.4708 (14)	C6B—C7B	1.4668 (14)
C7A—C8A	1.3895 (13)	C7B—C8B	1.3920 (13)
C8A—C9A	1.4233 (14)	C8B—C9B	1.4221 (14)
C8A—C10A	1.4978 (13)	C8B—C10B	1.4946 (14)
C10A—H10A	0.9600	C10B—H10D	0.9600
C10A—H10B	0.9600	C10B—H10E	0.9600
C10A—H10C	0.9600	C10B—H10F	0.9600
			
C7A—N1A—N2A	108.49 (8)	C7B—N1B—N2B	108.33 (8)
C7A—N1A—H1NA	129.6 (10)	C7B—N1B—H1NB	129.5 (11)
N2A—N1A—H1NA	121.6 (10)	N2B—N1B—H1NB	120.7 (11)
N1A—N2A—C9A	109.34 (8)	N1B—N2B—C9B	109.45 (9)
N1A—N2A—H2NA	123.7 (11)	N1B—N2B—H2NB	123.4 (10)
C9A—N2A—H2NA	125.5 (11)	C9B—N2B—H2NB	127.0 (10)
C2A—C1A—C6A	120.42 (10)	C2B—C1B—C6B	120.09 (10)
C2A—C1A—H1AA	119.8	C2B—C1B—H1BA	120.0
C6A—C1A—H1AA	119.8	C6B—C1B—H1BA	120.0
C1A—C2A—C3A	120.42 (10)	C1B—C2B—C3B	120.25 (10)
C1A—C2A—H2AA	119.8	C1B—C2B—H2BA	119.9
C3A—C2A—H2AA	119.8	C3B—C2B—H2BA	119.9
C2A—C3A—C4A	119.66 (11)	C4B—C3B—C2B	119.85 (11)
C2A—C3A—H3AA	120.2	C4B—C3B—H3BA	120.1
C4A—C3A—H3AA	120.2	C2B—C3B—H3BA	120.1
C5A—C4A—C3A	120.25 (10)	C5B—C4B—C3B	120.20 (10)
C5A—C4A—H4AA	119.9	C5B—C4B—H4BA	119.9
C3A—C4A—H4AA	119.9	C3B—C4B—H4BA	119.9
C4A—C5A—C6A	120.49 (10)	C4B—C5B—C6B	120.22 (10)
C4A—C5A—H5AA	119.8	C4B—C5B—H5BA	119.9
C6A—C5A—H5AA	119.8	C6B—C5B—H5BA	119.9
C5A—C6A—C1A	118.74 (10)	C5B—C6B—C1B	119.37 (10)
C5A—C6A—C7A	120.29 (9)	C5B—C6B—C7B	120.73 (9)
C1A—C6A—C7A	120.89 (9)	C1B—C6B—C7B	119.89 (9)
N1A—C7A—C8A	109.11 (9)	N1B—C7B—C8B	109.25 (9)
N1A—C7A—C6A	120.23 (9)	N1B—C7B—C6B	119.73 (9)
C8A—C7A—C6A	130.60 (9)	C8B—C7B—C6B	130.99 (9)
C7A—C8A—C9A	105.93 (8)	C7B—C8B—C9B	105.79 (8)
C7A—C8A—C10A	129.40 (9)	C7B—C8B—C10B	128.55 (10)
C9A—C8A—C10A	124.56 (9)	C9B—C8B—C10B	125.63 (9)
O1A—C9A—N2A	122.59 (9)	O1B—C9B—N2B	121.99 (9)
O1A—C9A—C8A	130.31 (9)	O1B—C9B—C8B	130.90 (9)
N2A—C9A—C8A	107.10 (9)	N2B—C9B—C8B	107.09 (9)
C8A—C10A—H10A	109.5	C8B—C10B—H10D	109.5
C8A—C10A—H10B	109.5	C8B—C10B—H10E	109.5
H10A—C10A—H10B	109.5	H10D—C10B—H10E	109.5
C8A—C10A—H10C	109.5	C8B—C10B—H10F	109.5
H10A—C10A—H10C	109.5	H10D—C10B—H10F	109.5
H10B—C10A—H10C	109.5	H10E—C10B—H10F	109.5
			
C7A—N1A—N2A—C9A	−1.33 (11)	C7B—N1B—N2B—C9B	−2.31 (11)
C6A—C1A—C2A—C3A	0.33 (18)	C6B—C1B—C2B—C3B	−0.54 (17)
C1A—C2A—C3A—C4A	0.55 (19)	C1B—C2B—C3B—C4B	0.32 (18)
C2A—C3A—C4A—C5A	−0.48 (18)	C2B—C3B—C4B—C5B	0.73 (17)
C3A—C4A—C5A—C6A	−0.47 (17)	C3B—C4B—C5B—C6B	−1.54 (16)
C4A—C5A—C6A—C1A	1.33 (16)	C4B—C5B—C6B—C1B	1.30 (15)
C4A—C5A—C6A—C7A	−175.25 (10)	C4B—C5B—C6B—C7B	−177.31 (9)
C2A—C1A—C6A—C5A	−1.26 (16)	C2B—C1B—C6B—C5B	−0.27 (15)
C2A—C1A—C6A—C7A	175.29 (10)	C2B—C1B—C6B—C7B	178.36 (10)
N2A—N1A—C7A—C8A	1.22 (11)	N2B—N1B—C7B—C8B	0.43 (11)
N2A—N1A—C7A—C6A	−176.28 (8)	N2B—N1B—C7B—C6B	178.45 (8)
C5A—C6A—C7A—N1A	141.18 (10)	C5B—C6B—C7B—N1B	139.78 (10)
C1A—C6A—C7A—N1A	−35.32 (14)	C1B—C6B—C7B—N1B	−38.83 (14)
C5A—C6A—C7A—C8A	−35.71 (16)	C5B—C6B—C7B—C8B	−42.70 (16)
C1A—C6A—C7A—C8A	147.80 (11)	C1B—C6B—C7B—C8B	138.69 (11)
N1A—C7A—C8A—C9A	−0.64 (11)	N1B—C7B—C8B—C9B	1.50 (11)
C6A—C7A—C8A—C9A	176.51 (10)	C6B—C7B—C8B—C9B	−176.23 (10)
N1A—C7A—C8A—C10A	175.59 (10)	N1B—C7B—C8B—C10B	−176.60 (10)
C6A—C7A—C8A—C10A	−7.25 (18)	C6B—C7B—C8B—C10B	5.67 (18)
N1A—N2A—C9A—O1A	−178.72 (9)	N1B—N2B—C9B—O1B	−175.40 (9)
N1A—N2A—C9A—C8A	0.91 (11)	N1B—N2B—C9B—C8B	3.21 (11)
C7A—C8A—C9A—O1A	179.42 (10)	C7B—C8B—C9B—O1B	175.58 (10)
C10A—C8A—C9A—O1A	2.96 (17)	C10B—C8B—C9B—O1B	−6.24 (17)
C7A—C8A—C9A—N2A	−0.17 (11)	C7B—C8B—C9B—N2B	−2.85 (11)
C10A—C8A—C9A—N2A	−176.63 (9)	C10B—C8B—C9B—N2B	175.32 (10)

### Hydrogen-bond geometry (Å, °)

**Table:**

*D*—H···*A*	*D*—H	H···*A*	*D*···*A*	*D*—H···*A*
N1B—H1NB···O1A^i^	0.913 (17)	1.796 (17)	2.7001 (11)	170.0 (16)
N1A—H1NA···O1B	0.935 (19)	1.78 (2)	2.6987 (14)	165.9 (16)
N2A—H2NA···O1A^ii^	0.93 (2)	1.768 (19)	2.6917 (12)	173.9 (17)
N2B—H2NB···O1B^iii^	0.934 (18)	1.752 (18)	2.6850 (13)	177.0 (16)

Symmetry codes: (i) *x*+1/2, −*y*+1/2, *z*+1/2; (ii) −*x*+1/2, −*y*+1/2, −*z*; (iii) −*x*+1, *y*, −*z*+1/2.
